# Bis(propane-1,3-diaminium) hexa­fluoridoferrate(III) fluoride trihydrate

**DOI:** 10.1107/S1600536810018039

**Published:** 2010-05-22

**Authors:** Aymen Zouaghi, Amor BenAli, Vincent Maisonneuve, Marc Leblanc

**Affiliations:** a Unité de Recherche 99/UR12-30, Faculté des Sciences de Bizerte, 7021 Jarzouna, Tunisia; b Laboratoire des Oxydes et Fluorures - UMR 6010 CNRS, Institut de Recherche en Ingénierie Moléculaire et Matériaux, Fonctionnels, IRIM2F FR CNRS 2775, Faculté des Sciences et Techniques, Université du Maine, Avenue Olivier Messiaen, 72085 LE MANS Cedex 9, France

## Abstract

The asymmetric unit of the title iron hybrid fluoride, (C_3_H_12_N_2_)_2_[FeF_6_]F·3H_2_O, contains two propane-1,3-diamin­ium [(H_2_dap)^2+^] cations, an octa­hedral [FeF_6_]^3−^ anion, an isolated F^−^ anion and three water mol­ecules of solvation. Each [FeF_6_]^3−^ anion is surrounded by four separate hydrogen-bonded water mol­ecules in the equatorial sites and by five separate aminium cation donor groups. The axial F atoms are only involved in N—H⋯F hydrogen bonds, resulting in a three-dimensional structure.

## Related literature

For general background to hybrid fluorides, their synthesis and their applications, see: Ben Ali *et al.* (2007 ▶, 2009 ▶); Adil *et al.* (2007 ▶); Latroche *et al.* (2006 ▶); Rother *et al.* (1998 ▶), Bentrup *et al.* (1998 ▶). For F⋯N inter­actions, see: Steiner (1998 ▶). For bond-valence sum (BVS) calculations, see: Brese & O’Keeffe (1991 ▶).
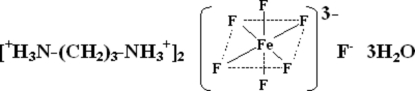


## Experimental

### 

#### Crystal data

(C_3_H_12_N_2_)_2_[FeF_6_]F·3H_2_O
*M* *_r_* = 395.18
Triclinic, 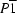

*a* = 9.844 (1) Å
*b* = 9.847 (1) Å
*c* = 10.7740 (8) Å
α = 106.959 (7)°
β = 95.379 (6)°
γ = 118.914 (9)°
*V* = 839.35 (17) Å^3^
*Z* = 2
Mo *K*α radiation
μ = 0.98 mm^−1^
*T* = 295 K
0.32 × 0.07 × 0.07 mm

#### Data collection

SIEMENS AED2 diffractometer
2920 measured reflections
2920 independent reflections
2599 reflections with *I* > 2σ(*I*)
3 standard reflections every 120 min  intensity decay: 4%

#### Refinement

*R*[*F* ^2^ > 2σ(*F* ^2^)] = 0.031
*wR*(*F* ^2^) = 0.093
*S* = 1.14
2920 reflections
219 parameters
H atoms treated by a mixture of independent and constrained refinement
Δρ_max_ = 0.42 e Å^−3^
Δρ_min_ = −0.35 e Å^−3^

### 

Data collection: *STADI4* (Stoe & Cie, 1998 ▶); cell refinement: *STADI4*; data reduction: *X-RED* (Stoe & Cie, 1998 ▶); program(s) used to solve structure: *SHELXS97* (Sheldrick, 2008 ▶); program(s) used to refine structure: *SHELXL97* (Sheldrick, 2008 ▶) within *WinGX* (Farrugia, 1999 ▶); molecular graphics: *DIAMOND* (Brandenburg & Putz, 2004 ▶) and *ORTEP-3* (Farrugia, 1997 ▶); software used to prepare material for publication: *enCIFer* (Allen *et al.*, 2004 ▶).

## Supplementary Material

Crystal structure: contains datablocks global, I. DOI: 10.1107/S1600536810018039/zs2036sup1.cif

Structure factors: contains datablocks I. DOI: 10.1107/S1600536810018039/zs2036Isup2.hkl

Additional supplementary materials:  crystallographic information; 3D view; checkCIF report

## Figures and Tables

**Table 1:** Hydrogen-bond geometry (Å, °)

*D*—H⋯*A*	*D*—H	H⋯*A*	*D*⋯*A*	*D*—H⋯*A*
N1—H1*B*⋯F2^i^	0.89	2.03	2.826 (3)	148
N1—H1*B*⋯F5^i^	0.89	2.22	2.839 (3)	127
N2—H2*A*⋯F4^ii^	0.89	1.82	2.672 (3)	161
N2—H2*B*⋯F7^ii^	0.89	1.85	2.735 (3)	172
N2—H2*C*⋯O1*W*^iii^	0.89	2.22	2.926 (3)	136
N2—H2*C*⋯F6^iii^	0.89	2.47	3.139 (3)	132
N3—H3*A*⋯F3^ii^	0.89	1.95	2.777 (3)	155
N3—H3*A*⋯F4^ii^	0.89	2.47	3.135 (3)	132
N3—H3*B*⋯F3^iv^	0.89	1.93	2.762 (3)	156
N4—H4*A*⋯F7^v^	0.89	1.86	2.728 (3)	164
N4—H4*B*⋯F6^vi^	0.89	2.09	2.886 (3)	149
N4—H4*B*⋯F1^vi^	0.89	2.33	3.029 (3)	135
O1*W*—H12⋯O3*W*^i^	0.81 (4)	1.99 (4)	2.787 (4)	173 (4)
O2*W*—H21⋯F1^vi^	0.76 (4)	1.91 (4)	2.606 (3)	153 (4)
O2*W*—H22⋯F6^i^	0.76 (4)	1.99 (4)	2.747 (3)	171 (4)
O3*W*—H32⋯O2*W*^vii^	0.73 (4)	2.04 (4)	2.766 (4)	173 (4)

Symmetry codes: (i) 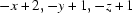; (ii) 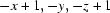; (iii) 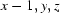; (iv) 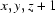; (v) 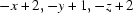; (vi) 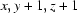; (vii) 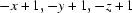.

**Fig. 1.**
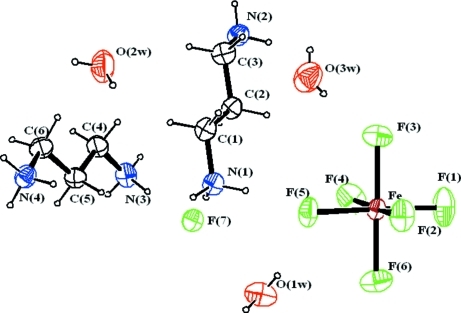
An view the (H2dap)2+ cation, the [FeF6]3- and F- anions and the water molecules of solvation (I). Thermal displacement ellipsoids are shown at the 50% probability level.

**Fig. 2.**
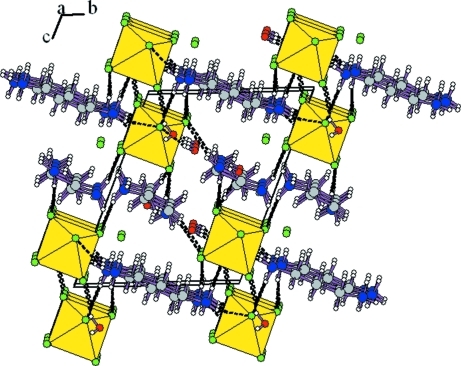
The three-dimensional H bonding network in the structure of (I).

**Fig. 3.**
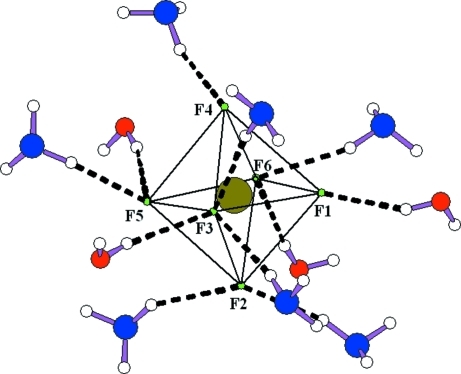
The environment of the FeF6 octahedron.

**Fig. 4.**
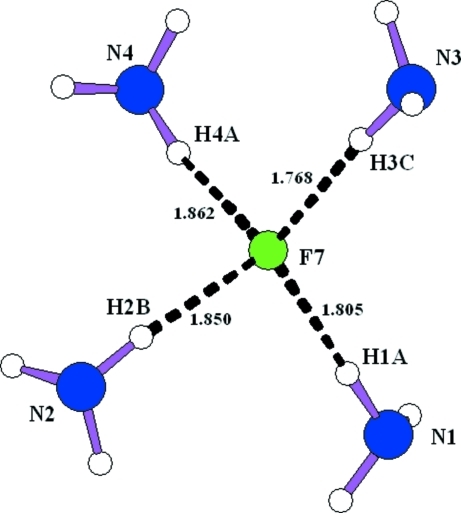
The environment of the isolated fluoride anion.

**Fig. 5.**
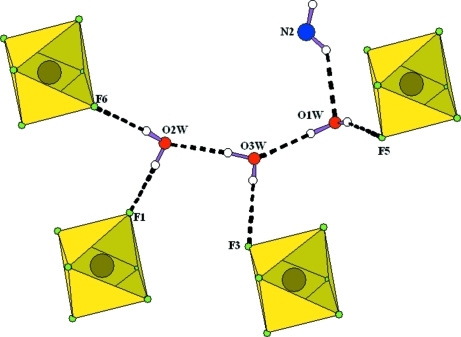
The environment of the water molecules

## supplementary crystallographic information

### Comment

The structure of the title compound (H_2_dap)_2_[FeF_6_](F).3H_2_O (I) consists of isolated FeF_6_ octahedra, diprotonated 1,3-diaminopropane (H_2_dap)^2+^ cations and three water molecules of solvation connected by a three-dimensional framework of hydrogen bonds in which isolated fluoride anions are located (Figure 1 and Figure 2). In (I) the [FeF_6_] complex anion adopts a slightly distorted octahedral environment, the Fe—F bond distance range [1.897 (2)–1.947 (2) Å] being typical of an octahedral iron^III^ environment. Each octahedral FeF_6_^3-^ anion is surrounded by four separate hydrogen-bonded water molecules in the equatorial sites and by seven separate aminium cation donor groups (Figure 3). The axial F atoms (F2, F4) are involved only in N–H···F interactions (Table 1). One of the equatorial F atom (F3), which has the longest Fe–F bond distance [1.947 (2)] Å), establishes three hydrogen bonds and consequently presents a low valence (0.47) with Fe^III^.

In fluoride metallates, "free" fluoride ions, are always surrounded by amine groups and their coordination number varies from 3 to 6. Also F···N distances increase with the coordination number (Steiner, 1998). In the title compound, "free" F ions adopt a tetrahedral coordination with four hydrogen atoms from four H_2_dap cations (Figure 4). The three hydrogen-bonded water molecules form trimer clusters, presenting various triangular environments with F acceptor atoms of the FeF_6_ octahedra and H donor atoms of the cation aminium groups (Figure 5). The infrared absorption spectrum of the title compound gives information on the organic moiety (C—C, C—N) and on the oxidation state of the iron atom, the presence of a vibrational band in the neighbourhood of 487 cm^-1^ being consistent with iron(III).

### Experimental

The title compound was prepared from a starting mixture of FeF_3_ (0.5 g) in 40% HF (3.0 ml) and ethanol (5 ml). 1,3-diaminopropane (2.7 ml) was added and mild hydrothermal conditions (463 K) were applied in a Teflon lined autoclave (25 ml). The resulting product was washed with ethanol and dried in air giving colourless single crystals.

### Refinement

All non-hydrogen atoms were refined with anisotropic displacement parameters. The H atoms of the water molecules were located using difference methods and their positional and isotropic displacement parameters were refined. Other H atoms including those on the aminium groups were included in the refinement at calculated positions and refined with a common isotropic thermal parameter.

### Crystal data

**Table:**

(C_3_H_12_N_2_)_2_[FeF_6_]F·3H_2_O	*Z* = 2
*M**_r_* = 395.18	*F*(000) = 414
Triclinic, *P*1	*D*_x_ = 1.564 Mg m^−^^3^
Hall symbol: -P 1	Mo *K*α radiation, λ = 0.71073 Å
*a* = 9.844 (1) Å	Cell parameters from 24 reflections
*b* = 9.847 (1) Å	θ = 5–20°
*c* = 10.7740 (8) Å	µ = 0.98 mm^−^^1^
α = 106.959 (7)°	*T* = 295 K
β = 95.379 (6)°	Parallelepiped, colorless
γ = 118.914 (9)°	0.32 × 0.07 × 0.07 mm
*V* = 839.35 (17) Å^3^	

### Data collection

**Table:**

SIEMENS AED2 diffractometer	*R*_int_ = 0.0000
Radiation source: fine-focus sealed tube	θ_max_ = 25.0°, θ_min_ = 2.1°
graphite	*h* = −11→11
2θ/ω scans	*k* = −10→11
2920 measured reflections	*l* = −12→0
2920 independent reflections	3 standard reflections every 120 min
2599 reflections with *I* > 2σ(*I*)	intensity decay: 4%

### Refinement

**Table:**

Refinement on *F*^2^	Primary atom site location: structure-invariant direct methods
Least-squares matrix: full	Secondary atom site location: difference Fourier map
*R*[*F*^2^ > 2σ(*F*^2^)] = 0.031	Hydrogen site location: inferred from neighbouring sites
*wR*(*F*^2^) = 0.093	H atoms treated by a mixture of independent and constrained refinement
*S* = 1.14	*w* = 1/[σ^2^(*F*_o_^2^) + (0.0485*P*)^2^ + 0.4679*P*] where *P* = (*F*_o_^2^ + 2*F*_c_^2^)/3
2920 reflections	(Δ/σ)_max_ < 0.001
219 parameters	Δρ_max_ = 0.42 e Å^−^^3^
0 restraints	Δρ_min_ = −0.35 e Å^−^^3^

### Special details

**Table:**

Geometry. All esds (except the esd in the dihedral angle between two l.s. planes) are estimated using the full covariance matrix. The cell esds are taken into account individually in the estimation of esds in distances, angles and torsion angles; correlations between esds in cell parameters are only used when they are defined by crystal symmetry. An approximate (isotropic) treatment of cell esds is used for estimating esds involving l.s. planes.
Refinement. Refinement of *F*^2^ against ALL reflections. The weighted *R*-factor wR and goodness of fit *S* are based on *F*^2^, conventional *R*-factors *R* are based on *F*, with *F* set to zero for negative *F*^2^. The threshold expression of *F*^2^ > σ(*F*^2^) is used only for calculating *R*-factors(gt) etc. and is not relevant to the choice of reflections for refinement. *R*-factors based on *F*^2^ are statistically about twice as large as those based on *F*, and *R*- factors based on ALL data will be even larger.

### Fractional atomic coordinates and isotropic or equivalent isotropic displacement parameters (Å^2^)

**Table:**

	*x*	*y*	*z*	*U*_iso_*/*U*_eq_	
Fe	0.84562 (4)	0.12759 (4)	0.22030 (3)	0.02722 (13)	
F1	0.7789 (3)	−0.0409 (2)	0.04636 (19)	0.0734 (6)	
F2	0.9740 (2)	0.29714 (19)	0.15238 (16)	0.0467 (4)	
F3	0.66528 (18)	0.1495 (2)	0.16599 (17)	0.0479 (4)	
F4	0.7093 (2)	−0.0330 (2)	0.28815 (18)	0.0582 (5)	
F5	0.91998 (19)	0.30634 (19)	0.39249 (15)	0.0460 (4)	
F6	1.0227 (2)	0.1036 (2)	0.2722 (2)	0.0582 (5)	
F7	0.79472 (17)	0.17081 (17)	0.74186 (14)	0.0363 (3)	
C1	0.6954 (3)	0.3991 (3)	0.6071 (2)	0.0330 (5)	
H1D	0.7029	0.4645	0.5530	0.0476 (17)*	
H1E	0.6899	0.4560	0.6948	0.0476 (17)*	
C2	0.5420 (3)	0.2254 (3)	0.5389 (3)	0.0327 (5)	
H2D	0.5468	0.1674	0.4512	0.0476 (17)*	
H2E	0.5322	0.1600	0.5935	0.0476 (17)*	
C3	0.3974 (3)	0.2417 (3)	0.5219 (3)	0.0371 (6)	
H3D	0.4063	0.3203	0.6061	0.0476 (17)*	
H3E	0.3969	0.2869	0.4531	0.0476 (17)*	
C4	0.6160 (3)	0.3197 (3)	0.9521 (3)	0.0371 (6)	
H4D	0.5557	0.3222	1.0181	0.0476 (17)*	
H4E	0.5624	0.3215	0.8729	0.0476 (17)*	
C5	0.7874 (3)	0.4733 (3)	1.0111 (3)	0.0336 (5)	
H5D	0.8430	0.4677	1.0868	0.0476 (17)*	
H5E	0.8456	0.4743	0.9431	0.0476 (17)*	
C6	0.7863 (3)	0.6330 (3)	1.0579 (3)	0.0368 (6)	
H6D	0.7111	0.6271	0.9884	0.0476 (17)*	
H6E	0.7498	0.6438	1.1388	0.0476 (17)*	
N1	0.8414 (2)	0.3911 (2)	0.6246 (2)	0.0318 (4)	
H1A	0.8289	0.3195	0.6639	0.0476 (17)*	
H1B	0.9268	0.4928	0.6762	0.0476 (17)*	
H1C	0.8567	0.3562	0.5442	0.0476 (17)*	
N2	0.2431 (2)	0.0780 (3)	0.4826 (2)	0.0368 (5)	
H2A	0.2398	0.0399	0.5484	0.0476 (17)*	
H2B	0.2363	0.0043	0.4069	0.0476 (17)*	
H2C	0.1607	0.0915	0.4689	0.0476 (17)*	
N3	0.6179 (3)	0.1637 (3)	0.9144 (2)	0.0393 (5)	
H3A	0.5179	0.0751	0.8720	0.0476 (17)*	
H3B	0.6561	0.1562	0.9887	0.0476 (17)*	
H3C	0.6809	0.1661	0.8599	0.0476 (17)*	
N4	0.9502 (3)	0.7830 (2)	1.0873 (2)	0.0347 (5)	
H4A	1.0194	0.7879	1.1504	0.0476 (17)*	
H4B	0.9471	0.8756	1.1168	0.0476 (17)*	
H4C	0.9820	0.7753	1.0123	0.0476 (17)*	
O1W	1.0878 (3)	0.2596 (3)	0.5849 (3)	0.0568 (6)	
O2W	0.6945 (3)	0.7508 (4)	0.7980 (3)	0.0660 (7)	
O3W	0.6382 (3)	0.4155 (3)	0.2944 (3)	0.0590 (6)	
H11	1.046 (6)	0.265 (6)	0.526 (5)	0.095 (18)*	
H12	1.168 (5)	0.352 (5)	0.625 (4)	0.067 (12)*	
H21	0.717 (5)	0.829 (5)	0.858 (4)	0.063 (12)*	
H22	0.772 (5)	0.782 (5)	0.776 (4)	0.064 (12)*	
H31	0.649 (5)	0.330 (4)	0.265 (4)	0.075 (12)*	
H32	0.550 (5)	0.373 (5)	0.277 (4)	0.054 (11)*	

### Atomic displacement parameters (Å^2^)

**Table:**

	*U*^11^	*U*^22^	*U*^33^	*U*^12^	*U*^13^	*U*^23^
Fe	0.02654 (19)	0.02225 (19)	0.02768 (19)	0.00942 (14)	0.01073 (14)	0.00871 (14)
F1	0.0992 (16)	0.0461 (10)	0.0417 (10)	0.0295 (11)	0.0150 (10)	−0.0069 (8)
F2	0.0495 (9)	0.0373 (8)	0.0437 (9)	0.0118 (7)	0.0234 (7)	0.0211 (7)
F3	0.0338 (8)	0.0473 (9)	0.0622 (10)	0.0206 (7)	0.0079 (7)	0.0244 (8)
F4	0.0472 (10)	0.0500 (10)	0.0602 (10)	0.0053 (8)	0.0147 (8)	0.0373 (9)
F5	0.0473 (9)	0.0379 (8)	0.0333 (8)	0.0151 (7)	0.0146 (7)	0.0019 (6)
F6	0.0478 (10)	0.0597 (11)	0.0797 (13)	0.0361 (9)	0.0190 (9)	0.0295 (10)
F7	0.0372 (8)	0.0356 (7)	0.0377 (8)	0.0180 (6)	0.0152 (6)	0.0176 (6)
C1	0.0341 (13)	0.0253 (11)	0.0363 (13)	0.0133 (10)	0.0121 (10)	0.0119 (10)
C2	0.0322 (13)	0.0263 (12)	0.0356 (13)	0.0135 (10)	0.0113 (10)	0.0103 (10)
C3	0.0329 (13)	0.0285 (12)	0.0463 (15)	0.0130 (11)	0.0113 (11)	0.0161 (11)
C4	0.0283 (12)	0.0379 (14)	0.0374 (13)	0.0118 (11)	0.0116 (10)	0.0150 (11)
C5	0.0281 (12)	0.0323 (13)	0.0375 (13)	0.0134 (11)	0.0099 (10)	0.0146 (11)
C6	0.0327 (13)	0.0388 (14)	0.0419 (14)	0.0190 (11)	0.0154 (11)	0.0181 (11)
N1	0.0287 (10)	0.0243 (10)	0.0363 (11)	0.0094 (8)	0.0116 (8)	0.0123 (8)
N2	0.0306 (11)	0.0338 (11)	0.0416 (12)	0.0155 (9)	0.0113 (9)	0.0122 (9)
N3	0.0316 (11)	0.0329 (11)	0.0376 (11)	0.0054 (9)	0.0138 (9)	0.0137 (9)
N4	0.0370 (11)	0.0281 (10)	0.0380 (11)	0.0173 (9)	0.0120 (9)	0.0112 (9)
O1W	0.0420 (13)	0.0576 (15)	0.0708 (16)	0.0241 (12)	0.0092 (11)	0.0318 (13)
O2W	0.0440 (14)	0.0710 (17)	0.0466 (13)	0.0213 (12)	0.0098 (11)	−0.0055 (13)
O3W	0.0513 (15)	0.0470 (13)	0.0641 (15)	0.0233 (12)	0.0159 (12)	0.0085 (11)

### Geometric parameters (Å, °)

**Table:**

Fe—F1	1.8968 (17)	C5—H5E	0.9700
Fe—F4	1.9083 (15)	C6—N4	1.487 (3)
Fe—F5	1.9157 (14)	C6—H6D	0.9700
Fe—F6	1.9234 (17)	C6—H6E	0.9700
Fe—F2	1.9405 (14)	N1—H1A	0.8900
Fe—F3	1.9468 (15)	N1—H1B	0.8900
C1—N1	1.475 (3)	N1—H1C	0.8900
C1—C2	1.519 (3)	N2—H2A	0.8900
C1—H1D	0.9700	N2—H2B	0.8900
C1—H1E	0.9700	N2—H2C	0.8900
C2—C3	1.508 (3)	N3—H3A	0.8900
C2—H2D	0.9700	N3—H3B	0.8900
C2—H2E	0.9700	N3—H3C	0.8900
C3—N2	1.483 (3)	N4—H4A	0.8900
C3—H3D	0.9700	N4—H4B	0.8900
C3—H3E	0.9700	N4—H4C	0.8900
C4—N3	1.480 (3)	O1W—H11	0.75 (5)
C4—C5	1.520 (3)	O1W—H12	0.81 (4)
C4—H4D	0.9700	O2W—H21	0.76 (4)
C4—H4E	0.9700	O2W—H22	0.76 (4)
C5—C6	1.511 (3)	O3W—H31	0.87 (4)
C5—H5D	0.9700	O3W—H32	0.73 (4)
			
F1—Fe—F4	92.30 (9)	C6—C5—C4	110.7 (2)
F1—Fe—F5	177.01 (8)	C6—C5—H5D	109.5
F4—Fe—F5	90.69 (8)	C4—C5—H5D	109.5
F1—Fe—F6	89.93 (10)	C6—C5—H5E	109.5
F4—Fe—F6	91.86 (8)	C4—C5—H5E	109.5
F5—Fe—F6	90.04 (8)	H5D—C5—H5E	108.1
F1—Fe—F2	89.21 (8)	N4—C6—C5	111.1 (2)
F4—Fe—F2	175.81 (8)	N4—C6—H6D	109.4
F5—Fe—F2	87.80 (7)	C5—C6—H6D	109.4
F6—Fe—F2	92.04 (8)	N4—C6—H6E	109.4
F1—Fe—F3	89.45 (9)	C5—C6—H6E	109.4
F4—Fe—F3	88.01 (8)	H6D—C6—H6E	108.0
F5—Fe—F3	90.59 (7)	C1—N1—H1A	109.5
F6—Fe—F3	179.36 (8)	C1—N1—H1B	109.5
F2—Fe—F3	88.10 (7)	H1A—N1—H1B	109.5
N1—C1—C2	112.05 (19)	C1—N1—H1C	109.5
N1—C1—H1D	109.2	H1A—N1—H1C	109.5
C2—C1—H1D	109.2	H1B—N1—H1C	109.5
N1—C1—H1E	109.2	C3—N2—H2A	109.5
C2—C1—H1E	109.2	C3—N2—H2B	109.5
H1D—C1—H1E	107.9	H2A—N2—H2B	109.5
C3—C2—C1	109.6 (2)	C3—N2—H2C	109.5
C3—C2—H2D	109.7	H2A—N2—H2C	109.5
C1—C2—H2D	109.7	H2B—N2—H2C	109.5
C3—C2—H2E	109.7	C4—N3—H3A	109.5
C1—C2—H2E	109.7	C4—N3—H3B	109.5
H2D—C2—H2E	108.2	H3A—N3—H3B	109.5
N2—C3—C2	112.1 (2)	C4—N3—H3C	109.5
N2—C3—H3D	109.2	H3A—N3—H3C	109.5
C2—C3—H3D	109.2	H3B—N3—H3C	109.5
N2—C3—H3E	109.2	C6—N4—H4A	109.5
C2—C3—H3E	109.2	C6—N4—H4B	109.5
H3D—C3—H3E	107.9	H4A—N4—H4B	109.5
N3—C4—C5	110.4 (2)	C6—N4—H4C	109.5
N3—C4—H4D	109.6	H4A—N4—H4C	109.5
C5—C4—H4D	109.6	H4B—N4—H4C	109.5
N3—C4—H4E	109.6	H11—O1W—H12	106 (5)
C5—C4—H4E	109.6	H21—O2W—H22	101 (4)
H4D—C4—H4E	108.1	H31—O3W—H32	100 (4)

### Hydrogen-bond geometry (Å, °)

**Table:**

*D*—H···*A*	*D*—H	H···*A*	*D*···*A*	*D*—H···*A*
N1—H1B···F2^i^	0.89	2.03	2.826 (3)	148
N1—H1B···F5^i^	0.89	2.22	2.839 (3)	127
N2—H2A···F4^ii^	0.89	1.82	2.672 (3)	161
N2—H2B···F7^ii^	0.89	1.85	2.735 (3)	172
N2—H2C···O1W^iii^	0.89	2.22	2.926 (3)	136
N2—H2C···F6^iii^	0.89	2.47	3.139 (3)	132
N3—H3A···F3^ii^	0.89	1.95	2.777 (3)	155
N3—H3A···F4^ii^	0.89	2.47	3.135 (3)	132
N3—H3B···F3^iv^	0.89	1.93	2.762 (3)	156
N4—H4A···F7^v^	0.89	1.86	2.728 (3)	164
N4—H4B···F6^vi^	0.89	2.09	2.886 (3)	149
N4—H4B···F1^vi^	0.89	2.33	3.029 (3)	135
O1W—H12···O3W^i^	0.81 (4)	1.99 (4)	2.787 (4)	173 (4)
O2W—H21···F1^vi^	0.76 (4)	1.91 (4)	2.606 (3)	153 (4)
O2W—H22···F6^i^	0.76 (4)	1.99 (4)	2.747 (3)	171 (4)
O3W—H32···O2W^vii^	0.73 (4)	2.04 (4)	2.766 (4)	173 (4)

Symmetry codes: (i) −*x*+2, −*y*+1, −*z*+1; (ii) −*x*+1, −*y*, −*z*+1; (iii) *x*−1, *y*, *z*; (iv) *x*, *y*, *z*+1; (v) −*x*+2, −*y*+1, −*z*+2; (vi) *x*, *y*+1, *z*+1; (vii) −*x*+1, −*y*+1, −*z*+1.
